# An immune cell activation signature reflected hepatocellular carcinoma heterogeneity and predicted clinical outcomes

**DOI:** 10.3389/fimmu.2025.1534611

**Published:** 2025-04-28

**Authors:** Xiaofeng Wang, Dongli Liu, Shuai Wang, Rui He

**Affiliations:** ^1^ Department of Medical Oncology, The First Affiliated Hospital of Zhengzhou University, Zhengzhou, Henan, ;China; ^2^ Department of Radiation Oncology, Shanghai General Hospital, Shanghai Jiao Tong University School of Medicine, Shanghai, ;China; ^3^ Department of Radiation, Chushi Orthopedic Hospital, Zhengzhou, Henan, ;China

**Keywords:** HCC, immune cell activation, single-cell sequencing, prognosis, microenvironment

## Abstract

**Background:**

The prognosis of hepatocellular carcinoma (HCC) remains challenging, and immune activation plays a critical role in cancer treatment. Identifying reliable immune activation-related prognostic markers is critical for predicting HCC patient outcomes.

**Method:**

A six-gene signature was developed. The prognostic value was assessed by correlating the signature and survival. The robustness of the signature was validated in three independent Gene Expression Omnibus (GEO) datasets. Associations with clinical, genomic, and transcriptomic features were also evaluated. Additionally, single-cell sequencing data were analyzed to explore cell–cell interaction heterogeneity reflected by the signature. The biological role of candidate gene RORC was investigated, including chemotherapy resistance and detailed regulatory mechanism in affecting progression. The clinical potential role of RORC and its downstream gene was also evaluated by immunohistochemical (IHC) microarray.

**Results:**

The six-gene signature stratified patients into high-risk and low-risk groups, with high-risk samples exhibiting significantly shorter overall survival (median: 23.8 months, 95% CI: 20.6–41.8) than low-risk samples (median: 83.2 months, 95% CI: 69.6–NA, p < 0.001). Validation in independent GEO datasets confirmed the robustness of the signature. The signature was significantly associated with the pathological stage and negatively correlated with PD-L1 expression, outperforming clinical indicators in predicting 3-year survival. The signature was significantly associated with TP53 mutations, genomic stability, and canonical cancer-related pathways. Single-cell sequencing data indicated that the signature revealed cell–cell interaction heterogeneity in HCC. Candidate gene RORC promotes proliferation and migration by regulating CDC6 gene expression as a transcription factor. Furthermore, RORC is also associated with multiple drug resistance, especially docetaxel and paclitaxel. IHC revealed that RORC and candidate gene CDC6 were valuable predictive biomarkers for prognosis.

**Conclusion:**

The six-gene signature provides valuable insights into the biological status of HCC patients and is a robust tool for clinical application.

## Introduction

Hepatocellular carcinoma (HCC) is among the most common cancers and is the third leading cause of cancer-related deaths worldwide. The incidence of HCC is expected to exceed 1,000,000 cases by 2025 (1, 2). However, early diagnosis occurs in fewer than 20% of HCC patients, with over 50% diagnosed at advanced stages and more than 70% facing recurrence within 5 years after first-line treatment (3, 4). HCC is among the most heterogeneous cancers and is characterized by multiple factors, including biological (genetic mutation, transcriptome, etc.), clinical (staging, exposure, etc.), and microenvironmental (immune cell infiltration, activation, etc.) status (5, 6), which makes it difficult to predict the clinical outcome of HCC patients.

Despite the efforts devoted to screening effective biomarkers for HCC in recent decades, the reproducibility of these methods is still unsatisfactory. For example, Qi et al. reported that aberrant PTTG1 gene expression increases asparagine production and thus regulates mTOR activity to promote the progression of HCC (7). Similarly, LYZ was shown to stimulate the STAT3 signaling pathway and is a biomarker for the progression phenotype of HCC (8). Using paired normal and tumor tissue and immunohistochemical staining, Yi-Chieh reported that TMED9 may be used as a predictive biomarker and potential treatment target (9), which was similar to the other cancer-related genes in other cancer types (10, 11). However, HCC heterogeneity limits the clinical utilization of these biomarkers, and the performance of these markers has not been validated across centers. To address this issue, multiple genes/omic signatures were recently developed and emphasized. Early in 2002, Laura et al. developed a 70-gene signature to predict the clinical outcome of patients with breast cancer (12). After retrospective randomized validation in 6,693 women across centers, the “mammaprint” signature was shown to be a powerful biomarker for assessing ER+/HER2− early-stage breast cancer (13) radiotherapy necessity. Similarly, Oncotype DX and other signatures have also been reported (14). In HCC, Hao et al. constructed a signature using T-cell exhaustion genes and bulk gene expression data from The Cancer Genome Atals (TCGA) and single-cell sequencing (15). Similarly, using pyroptosis-related genes, a signature for predicting the outcome of HCC patients was also developed (16). The signatures were effective in predicting the survival of patients with HCC in TCGA dataset, but their robustness and reproducibility are still unknown. Furthermore, the functions of the specified genes used for the models mentioned above are unclear.

As a hallmark of cancers, immune escape is a necessary step, including HCC genesis (17). Immune suppression is critical for HCC genesis, and immune cell activation is critical for treatment, especially immunotherapy targeting PD-1/PD-L1/CTLA4 (1). For example, Marina et al. reported that the activation of beta-catenin, the core gene in the WNT signaling pathway, facilitated immune escape and resistance to immunotherapy in HCC (18). To this end, by analyzing and optimizing immune cell activation-related genes, a signature based on six genes was constructed and validated. The heterogeneity reflected by the signature was analyzed, including mutation, transcription, immune infiltration, drug response, and clinical indicators. Single-cell sequencing revealed that the signature was significantly associated with cell infiltration and the cell–cell interaction profile. The function and mechanism of candidate gene RORC were also investigated. These results indicate that the signature is a robust and reproducible marker for predicting the clinical outcome of HCC patients.

## Materials and methods

### Raw data retrieval and data processing

The gene expression, mutation, copy number variation, and clinical information of TCGA dataset were retrieved from UCSC Xena (19) and cBioPortal (20). The expression matrices of GSE14520, GSE36376, and GSE77314 were downloaded from the Gene Expression Omnibus (GEO) database, along with their corresponding survival information. The gene expression data of TCGA and GSE77314 were in the form of log2-transformed RSEM counts for further analyses. Background correction and normalization: For the data from GSE14520 and GSE36376, background correction was carried out using the robust multi-array average (RMA) for arrays from both Affymetrix and Illumina. Subsequently, normalization was performed using quantile normalization. Probe annotation: After microarray data normalization, probes were annotated using a manufacturer-provided reference file for GPL571 (Affymetrix Human Genome U133A 2.0 Array) and GPL10558 (Illumina HumanHT-12 V4.0 expression beadchip). For probes matching more than one gene, the probes were omitted. If several probes matched one gene, only the probe with the highest mean signaling intensity across samples in the dataset was retained. This process was implemented using an in-house R script for data manipulation and comparison of probe intensities.

### Candidate gene selection and signature construction

The Gene Ontology gene list “CELL ACTIVATION INVOLVED IN IMMUNE RESPONSE” term was downloaded from the MSIGDB database (21). Cox univariate regression: In TCGA dataset, Cox univariate regression analysis was performed using the coxph function from the R package “survival”. The formula for the univariate regression was Surv(time, event) ~ gene_expression, where time represents the survival time, event indicates the event status (e.g., death), and gene_expression is the expression level of each gene in the gene list. The significance level for this analysis was set at α = 0.05. Grouped survival difference analyses: Survival data in TCGA dataset were grouped based on the median expression level of each gene. The high/low-expression group was defined as the expression level higher/lower than the median of the samples. The Kaplan–Meier survival curves were plotted for the high- and low-expression groups, and the log-rank test was used to assess the significance of survival differences between the two groups. In this step, the R package “survival” was used. Genes that were significantly associated with overall survival according to both Cox univariate regression (p < 0.05) and grouped survival difference analyses (p < 0.05) were retained as candidate genes. Panel optimization: Panel optimization was carried out by enumerating all combinations of candidate genes (gene number <7) using the combn function in R. In this step, all possible combinations of the candidate genes selected were listed and used for further analysis. For each combination, a Cox multivariate regression model was constructed in TCGA dataset using the coxph function with the formula Surv(time, event) ~ combination_of_genes. The sample datasets were equally divided into high- and low-risk groups based on the risk scores calculated from the Cox multivariate regression model. Survival differences between the high- and low-risk groups were evaluated using the log-rank test as described above, and p-values for all combinations were recorded. The combination with the smallest p-value from the log-rank test was retained as the optimized panel. Survival analysis was performed using the R package “survival”, and gene expression was visualized using “pheatmap”.

### Signature validation and clinical associations

The signature scores of the GSE14520, GSE36376, and GSE77314 datasets were calculated using the same formula used for the training dataset. The samples were also equally separated into low- and high-risk samples according to the corresponding median values. For clinical information, risk score differences were calculated using Student’s t-test and visualized using the R package “vioplot”. The results of Cox multivariate regression were visualized using “survminer”. Based on the relevant data in TCGA and the model, the nomogram and calibration curve were generated and visualized using “rms”.

### Genomic and transcriptome associations

Non-synonymous mutations in TCGA dataset were used to construct a mutation matrix. The differences in mutations between the high- and low-risk samples were evaluated using Fisher’s exact test, and the mutational landscape was visualized using the R package “GenVisR” (22). Copy number differences were evaluated using the Wilcoxon rank sum test and visualized using “pheatmap”. The differentially expressed genes between groups were identified using the R package “limma” with an adjusted p-value <0.01 and a |log2-fold change| > 2. The significantly enriched Gene Ontology (molecular function, cellular component, and biological process) and Kyoto Encyclopedia of Genes and Genomes (KEGG) pathway terms were evaluated and visualized using the R package “clusterProfiler” (23). For gene set enrichment analyses, the genes were ranked by fold change and visualized using default parameters.

### Cell infiltration and drug response

Infiltration was estimated using different algorithms based on the transcriptome of TCGA dataset, including XCELL (24), CIBERSORT (25), TIMER (26), QUANTISEQ (27), EPIC (28), and TIMER2.0. Differences in infiltration between groups were quantified using Student’s t-test and visualized using the R packages “vioplot” and “pheatmap”. Similarly, the IC50 values of each TCGA sample were quantified using gene expression values and a default training dataset with the R package “OncoPredict” (29). The differences in the IC50 values were also determined and visualized.

### Single-cell sequencing data processing and cell–cell interactions

The single-cell sequencing R data for 14 processed HCC patients were retrieved from the GEO database (accession number GSE156625). The data were processed according to a previously reported method (30), with the annotation results. The data were processed using the R package “Seurat” (31). Pseudo RNA-seq data were generated using Seurat::AggregateExpression, the expression data were log2 transformed to calculate the risk score values of these 14 samples, and the cell proportions were subsequently visualized. The R packages “CellChat” (32) and “Patchwork” were used for intercellular communication analysis with default parameters.

### CCK-8 and migration assay

The cells were treated with trypsin, resuspended in a complete medium, and counted. For each group, 2,000 cells per well in 100 µL of medium were seeded in triplicate. The cells were allowed to fully settle, the cell density was observed, and the cells were cultured. Starting from the second day after plating, 10 µL of CCK-8 solution was added to each well. After 1–3 hours, the plate was gently shaken for 2–5 minutes, and a spectrophotometer was used to measure the optical density (OD) at 450 nm.

Chambers were placed into an empty 24-well plate, 100 µL of serum-free medium was added to the chambers, and the chambers were hydrated for 1–2 hours. The cells were digested with trypsin, suspended, and counted. The medium was removed, and 600 µL of medium containing 30% fetal bovine serum (FBS) was added to the lower chamber. The cell suspension was diluted with serum-free medium, and 100 µL of the suspension was added to each chamber. The chambers were transferred into a medium containing 30% FBS and incubated for 4–24 hours. The medium was removed; the cells were fixed and incubated at room temperature for 10–30 minutes. The upper chamber was washed with 1× Phosphate Buffered Saline (PBS), the cells were stained by immersing them in a staining solution for 5–10 minutes, the excess stain was absorbed, and non-migratory cells were removed, washed again, air-dried, and photographed.

### ChIP-PCR and luciferase assay

Chromatin immunoprecipitation (ChIP) was conducted utilizing a ChIP assay kit (Millipore Sigma, Burlington, MA, USA) following the manufacturer’s instructions. The eluted DNA was immunoprecipitated with either an IgG or an anti-RORC antibody (14-6988-82, eBioscience, San Diego, CA, USA). Quantification of each immunoprecipitated DNA sample was performed via quantitative PCR (qPCR) using primers specifically designed to amplify the proximal promoter region of the CDC6 gene, encompassing predicted RORC binding sites. All assays were conducted in triplicate, and the results were normalized to the input DNA.

The CDC6 3′UTR was cloned into the psi-CHECK2 vector and transfected into the cells using Lipofectamine 3000 under standard conditions at 37°C for 4 hours. Luciferase activity was assessed after 48 hours of incubation using the dual-luciferase reporter assay system (Promega, Madison, WI, USA) with measurements taken at 490 nm. Firefly luciferase activity was normalized by calculating the ratio of firefly to Renilla luciferase activity. The primer sequences were listed as follows: RORC-F: GTAACGCGGCCTACTCCTG, RORC-R: GTCTTGACCACTGGTTCCTGT; GAPDH-F: ACAACTTTGGTATCGTGGAAGG, GAPDH-R: GCCATCACGCCACAGTTTC; CDC6-F: AAGCCCTG, CDC6-R: TCAAATACCAATCTTCGTCCC. Sequences of siCDC6 were 5′-AGACUAUAACUCUACAGAUUGUGdAdA-3′. The siRORC sequences were siRORC-1 (UUAAAGUGCACAUAAGAGGUUCCUCUUAUGUGCACUUUAAAG), siRORC-2 (UUUAAAGUGCACAUAAGAGGUCUCUUAUGUGCACUUUAAAGA), siRORC-3 (UCUUUAAAGUGCACAUAAGAGCUUAUGUGCACUUUAAAGAUA).

### Immunohistochemistry

Primary anti-CDC6 antibody (1:500; 11640‐1‐AP; Proteintech, Chicago, IL, USA) and anti-RORC antibody (1:500, 14-6988-82, eBioscience) were utilized for immunohistochemical (IHC) staining. At least three pathologists not informed of the clinical, pathological, or clinical outcome of all patients participated in quantifying the signal intensity of the samples. The samples were deparaffinized, hydrated, blocked, and added into the primary anti-CDC6 rabbit antibody (diluted 1:500) and cultured for 10 h at 4°C.

## Results

### Gene screening and signature construction

**Figure 1:**
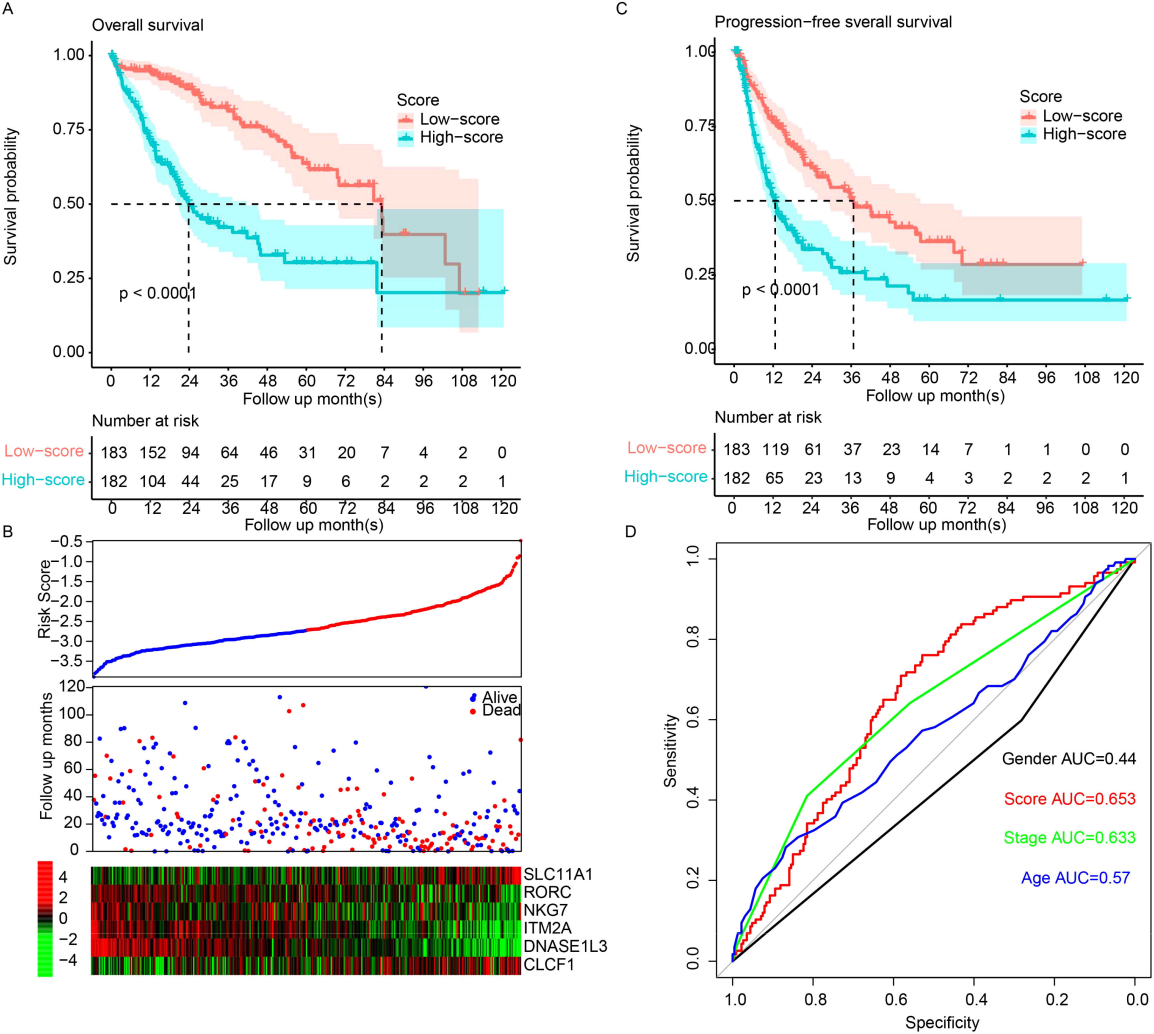
The model predicts HCC survival. **(A)** Overall survival in high-risk samples was significantly worse than that in low-risk samples. **(B)** Low-risk samples show high expression of tumor suppressor genes and low expression of oncogenes. TCGA samples were sorted by scores from low to high, and overall survival and candidate gene abundances were visualized using “pheatmap”. **(C)** Progression-free survival demonstrated a similar pattern. **(D)** The Area Under the Receiver Operating Characteristic (AUROC) for overall survival was calculated for both clinical indicators and the model. HCC, hepatocellular carcinoma.

The correlation between the expression level of the immune cell activation gene and overall survival in TCGA dataset was estimated using Cox univariate regression, and survival differences between the high-expression and low-expression groups were determined. In total, 22 genes were identified. To facilitate clinical practice and remove redundant information, combinations of genes with fewer than six genes were enumerated. Signatures were constructed by applying Cox multivariate regression on different gene combinations. The combination with the smallest p-value was identified as the optimized panel and signature. As a result, a signature based on six genes (SLC11A1, RORC, NKG7, ITM2A, DNASE1L3, and CLCF1) was constructed. The signature values were calculated as follows: signature = (0.07577395 * SLC11A1) + (−0.15675603 * RORC) + (−0.18440305 * NKG7) + (−0.01589646 * ITM2A) + (−0.08330426 * DNASE1L3) + (0.07315706 * CLCF1). The samples were divided into the high- and low-risk groups, and the median value across TCGA samples was used as the cutoff. The high-risk samples were characterized by significantly shorter survival (median survival: 23.8 months, 95% CI: 20.6–41.8) than the low-risk samples (median: 83.2, 95% CI: 69.6–NA, p < 0.001) (**Figure 1A**). SLC11A1 and CLCF1 were overexpressed in the high-risk samples, while RORC, NKG7, ITM2A, and DNASE1L3 were more highly expressed in the low-risk samples (**Figure 1B**). Consistent with this finding, the high-risk samples also had a significantly worse progression-free survival rate than the low-risk samples (**Figure 1C**). The 3-year survival ROC curve was visualized (**Figure 1D**), and the area under the curve (AUC) was calculated. Compared to clinical indicators, including age, sex, and stage, the signature performed better. Collectively, these results suggest that the signature is a valuable biomarker for predicting the prognosis of HCC patients.

### Signature validation using independent datasets

**Figure 2:**
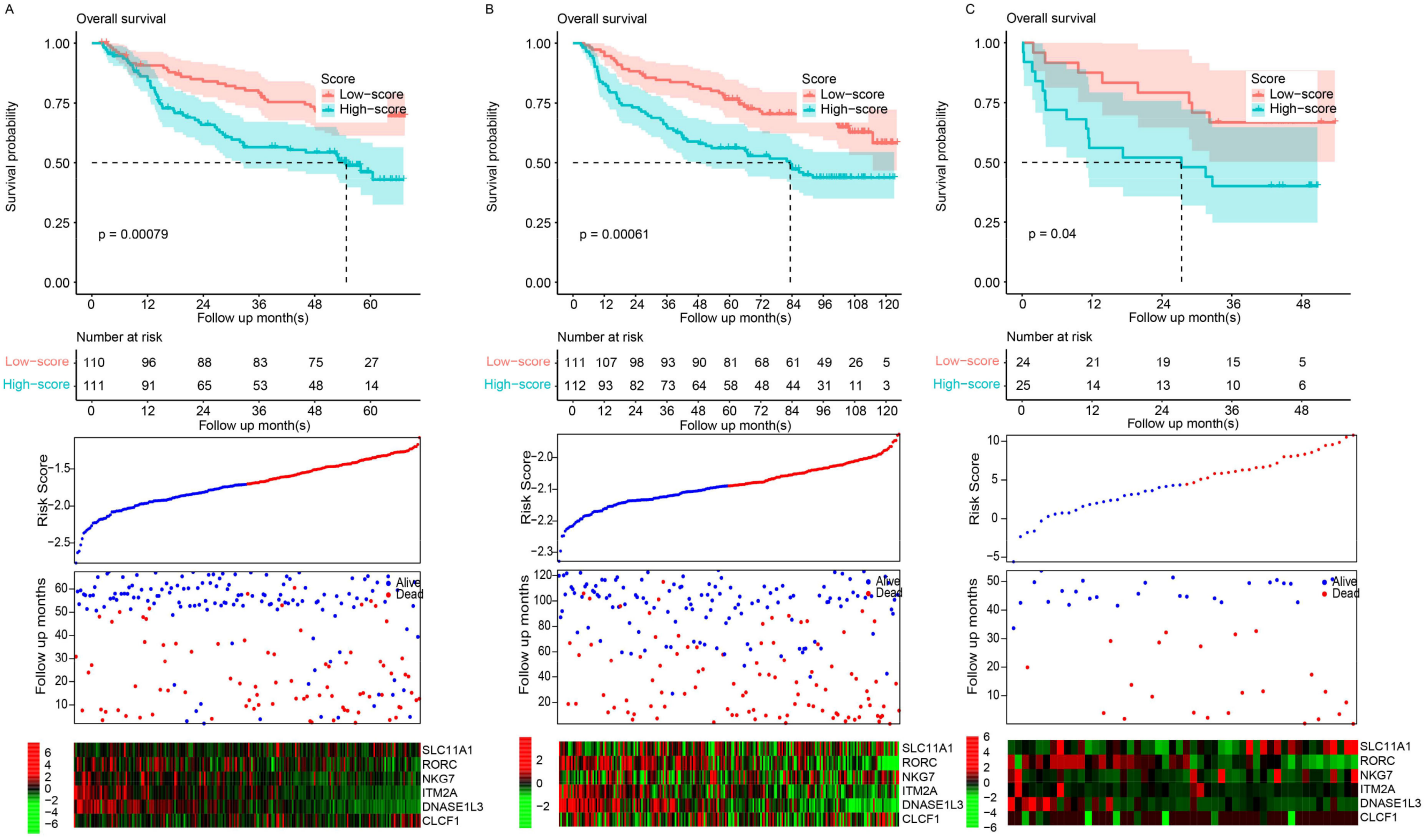
Model validation. Scores were evaluated, and datasets were classified into high- and low-risk groups based on the median score. Overall survival patterns between these groups were evaluated in the GSE14520 **(A)**, GSE36376 **(B)**, and GSE77314 **(C)** datasets.

To verify the robustness of the model, it was validated across three entirely independent GEO datasets: GSE14520, GSE36376, and GSE77314. The signature score was calculated according to the expression values and the corresponding coefficients in the above formula. The samples in each dataset were also divided into the high- and low-risk groups based on the median value in each dataset. The high-risk samples had a significantly shorter survival period than the low-risk samples in the GSE14520 (**Figure 2A**), GSE36376 (**Figure 2B**), and GSE77314 (**Figure 2C**) datasets. Consistently, the gene expression patterns of the candidate genes also resembled those in the training dataset, TCGA (**Figures 2A–C**, bottom panel). In summary, the performance of the signature was robust and reproducible across datasets instead of being a result of overfitting.

### Signature and clinical indicators

**Figure 3:**
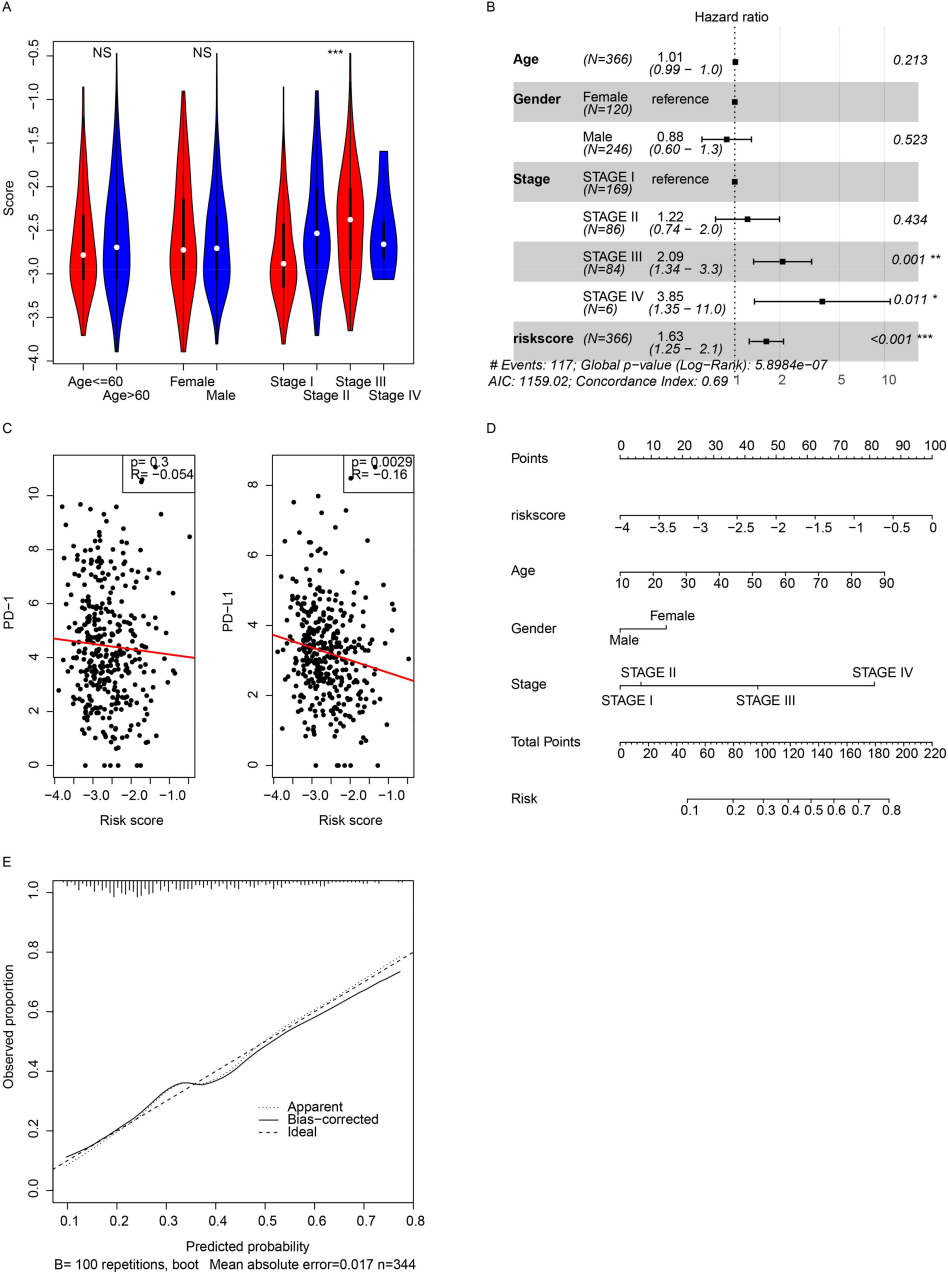
Clinical correlations. **(A)** Correlations between the score and pathological indicators were analyzed. **(B)** Cox multivariate regression was performed using the score and pathological indicators. **(C)** The model showed a significant association with PD-L1 gene expression. **(D)** A 3-year overall survival nomogram was created using clinical indicators and the model, with a calibration curve **(E)**. *, p<0.05, **, p<0.01, ***, p<0.001. NS, Not Significant.

The relationships between the signature and clinical indicators were evaluated. The signature was significantly associated with pathological stage but was independent of age and sex (**Figure 3A**). Cox multivariate regression revealed that the signature was significantly associated with survival (p < 0.001), while age and sex were not (**Figure 3B**). The immune status and immunotherapy biomarker PD-L1 were significantly negatively associated with the signature (**Figure 3C**), while PD-1 was not. A nomogram was constructed to predict the 3-year survival of patients with HCC in TCGA dataset (**Figure 3D**). The risk score had a broader range of points (from 0 to 100), indicating a greater impact on the total score compared to age, sex, and stage, which have narrower ranges. The calibration curve was also visualized (**Figure 3E**). These results indicate that the signature reflects some clinical indicators but contains more information for predicting the prognosis of HCC patients.

### Genomic signature of the model

**Figure 4:**
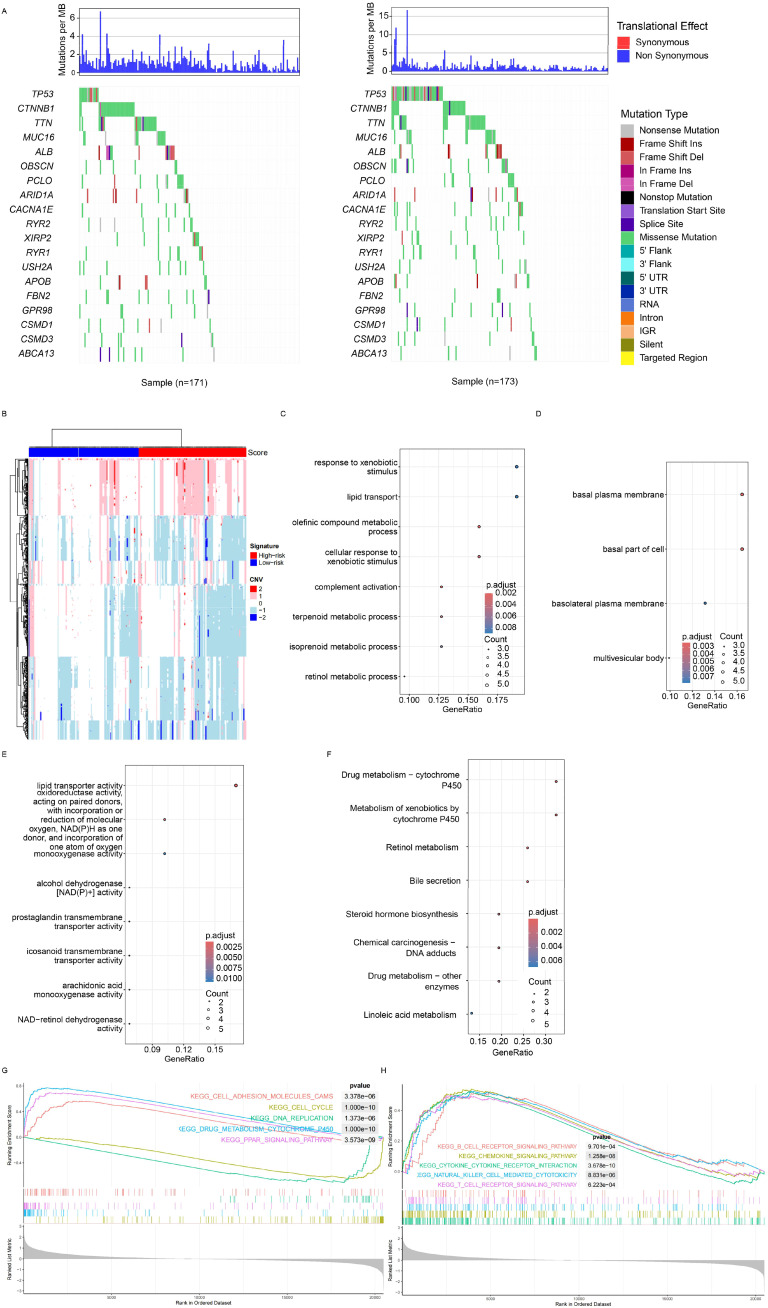
Genomic and transcriptomic features. **(A)** Specific highly mutated genes were identified for both the high- and low-risk groups. **(B)** Copy number variations were detected, with red and green bars indicating the high- and low-score groups, respectively. Differentially expressed genes (DEGs) between groups were identified, followed by enrichment analysis of Gene Ontology terms, including cancer-related biological processes **(C)**, molecular functions **(D)**, and cellular components **(E)**. **(F)** Gene set enrichment analysis revealed significant enrichment in HCC-related pathways, adhesion pathways **(G)**, and immune-related pathways **(H)**. HCC, hepatocellular carcinoma.

As mentioned, multiple genes/omic signatures may reflect various biological statuses of cancer, and the relationships between the signature and mutation, copy number variation (CNV), and transcriptome were investigated next. The mutation rate of TP53 was significantly different between the low- and high-risk groups, and the mutation rate was significantly higher in the high-risk group (**Figure 4A**, right panel). At the CNV level, variations were more frequent (**Figure 4B**) in the high-risk samples. Differentially expressed genes between the high- and low-risk groups were identified, and the enriched Gene Ontology (including molecular function, cellular component, and biological processes) was also identified (**Figures 4C–E**). Cancer progression-related processes and pathways were also identified. Gene Set Enrichment Analysis (GSEA) revealed that canonical cancer pathways were enriched in the high-risk group (**Figure 4F**). In addition to the cell cycle, cell adhesion, and drug metabolism pathways (**Figure 4G**), immune response-related pathways, including cytokine–cytokine interaction, chemokine signaling, and NK cell-mediated cytotoxicity, were also significantly enriched (**Figure 4H**). Overall, the signature reflected the biological status of HCC at both the genome and transcriptome levels.

### Immune infiltration and the signature

**Figure 5:**
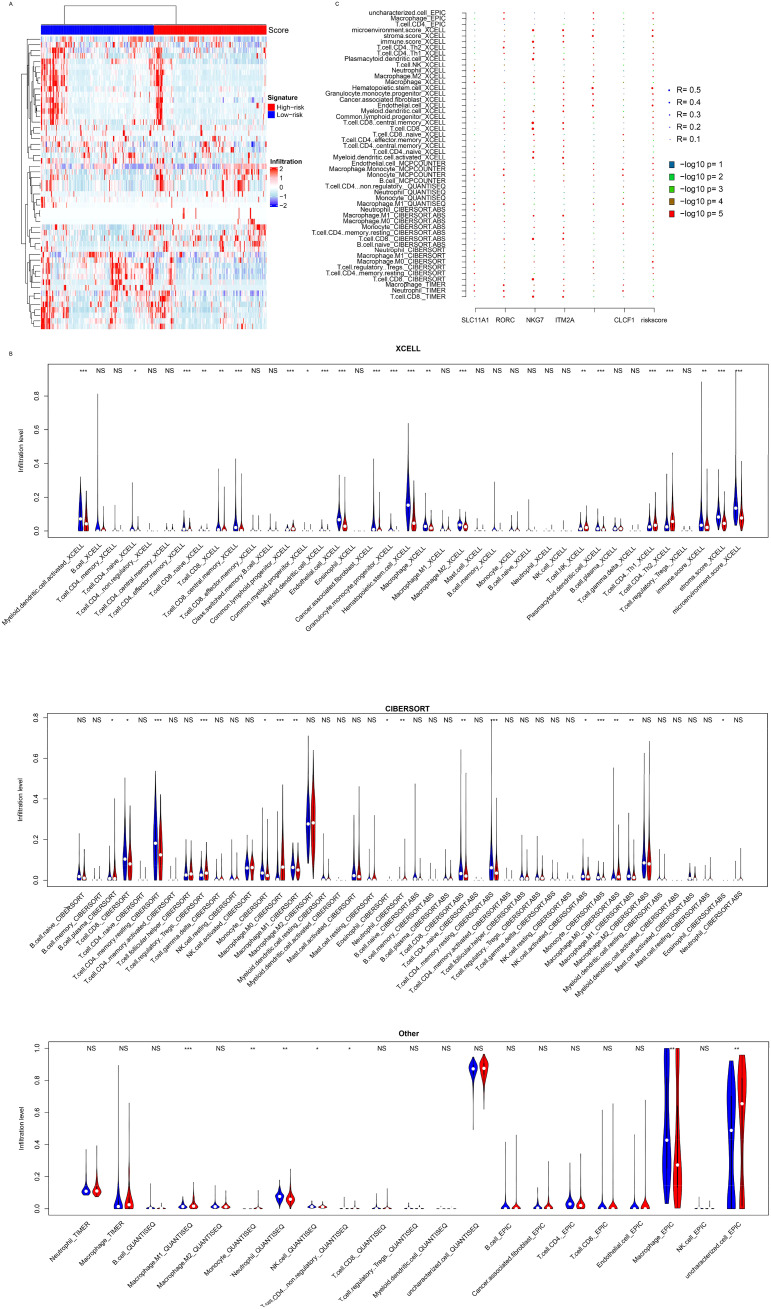
Immune infiltration. **(A)** The model is significantly associated with the infiltration of various immune cell types, as determined by algorithms such as CIBERSORT, XCELL, TIMER, EPIC, and MCPCOUNTER **(B)**. **(C)** Correlation analyses showed that candidate genes contributed to the signature associations. *, p<0.05, **, p<0.01, ***, p<0.001. NS, Not Significant.

The signature was significantly associated with various immune pathways and PD-L1 expression, and the signature was constructed with immune cell activation-related genes, which prompted us to analyze immune cell infiltration and the signature. Immune cell infiltration was estimated with multiple algorithms (including CIBERSORT, TIMER, XCELL, and EPIC) using the transcriptome of TCGA dataset. A high proportion of immune cells was differentially infiltrated according to these algorithms (**Figure 5A**). CD8+ T cells, M0 macrophages, and NK cells were differentially infiltrated between the low- and high-risk groups (**Figure 5B**). Compared to the candidate genes, the signature more significantly and comprehensively reflected immune infiltration (**Figure 5C**). Compared to the candidate genes, the signature was significantly correlated with all immune cell types listed, while the candidate genes were not. Collectively, the results above indicate that the signature represents the immune infiltration of HCC.

### Single-cell landscape and signature

**Figure 6:**
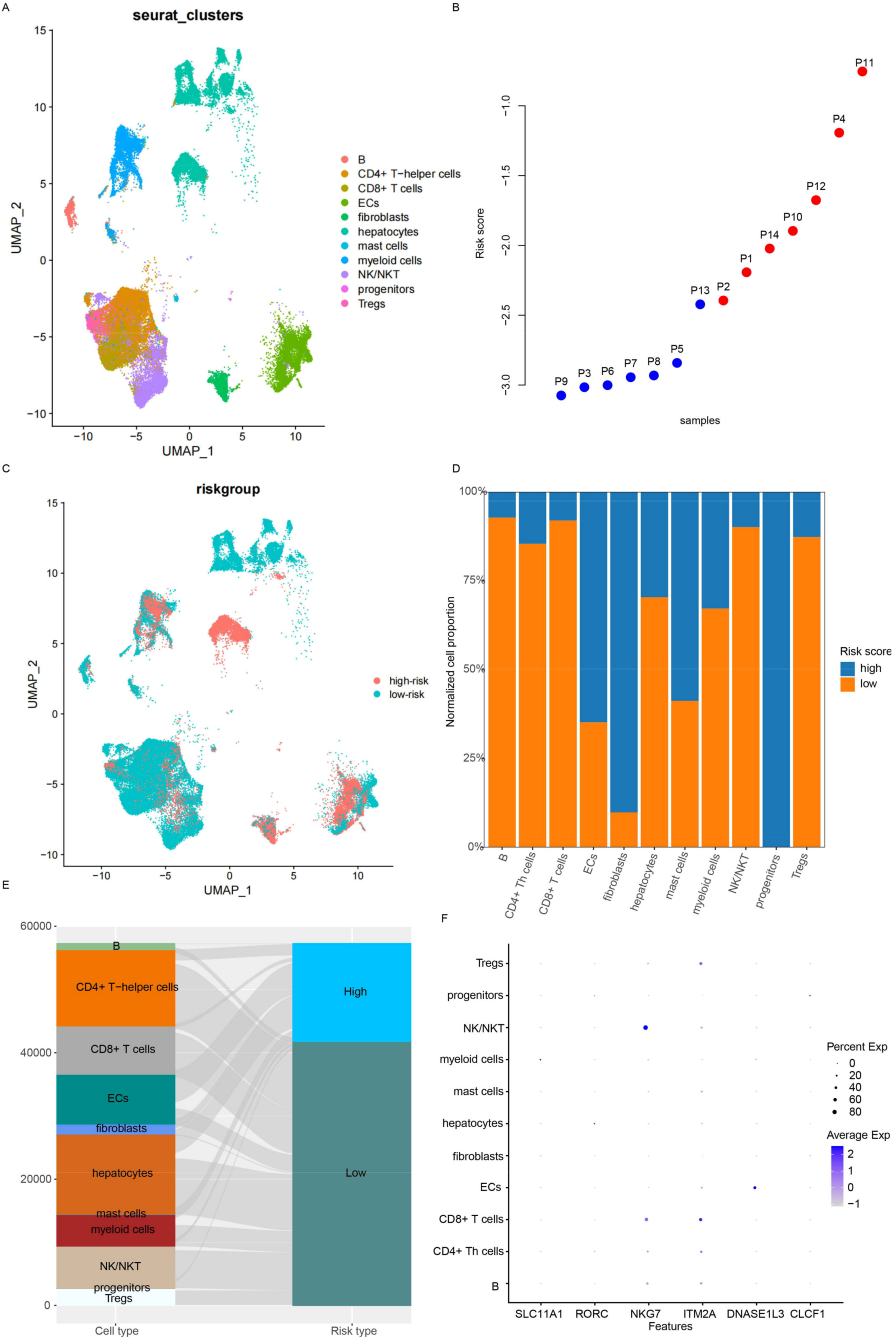
Microenvironment status at the single-cell level. **(A)** Cell type distribution was analyzed. **(B)** The risk score for each sample was calculated using pseudo bulk RNA-seq. **(C)** While the distribution of cell types was similar between low- and high-risk samples, the proportions differed, as visualized by bar plots **(D)** and Sankey diagrams **(E)**. **(F)** The expression of candidate genes across cell types was also examined.

The immune infiltration algorithms were based on the expression of cell lineage-specific genes, but the single-cell landscape is still vague. Single-cell sequencing data from 14 HCC samples (GSE156625) were used for analyses. After preprocessing and annotation (**Figure 6A**), the samples’ signature values were calculated using aggregated expression to generate pseudo bulk RNA-seq data. The signature values were calculated for 14 samples (**Figure 6B**), and these samples were divided into the high- and low-risk groups. Almost all cell types were distributed in the high- and low-risk groups (**Figure 6C**), but the cell proportions were different (**Figures 6D, E**). Importantly, immune activation cells, including Tregs and CD4+ and CD8+ T cells, were highly enriched in the low-risk group, while basal cells (Epithelial cells (ECs), fibroblasts, and hepatocytes) were more abundant in the high-risk group. The candidate genes used for signature construction were expressed in different cell types (**Figure 6F**). NKG7 was expressed in NK/NKT cells, ITM2A had the highest expression in CD8+ T cells, and DNASE1L3 was detected in ECs. These results indicate that the candidate genes originated from different cell types and that the signature reflected immune infiltration at the single-cell level.

### Cell–cell interaction and the signature

**Figure 7:**
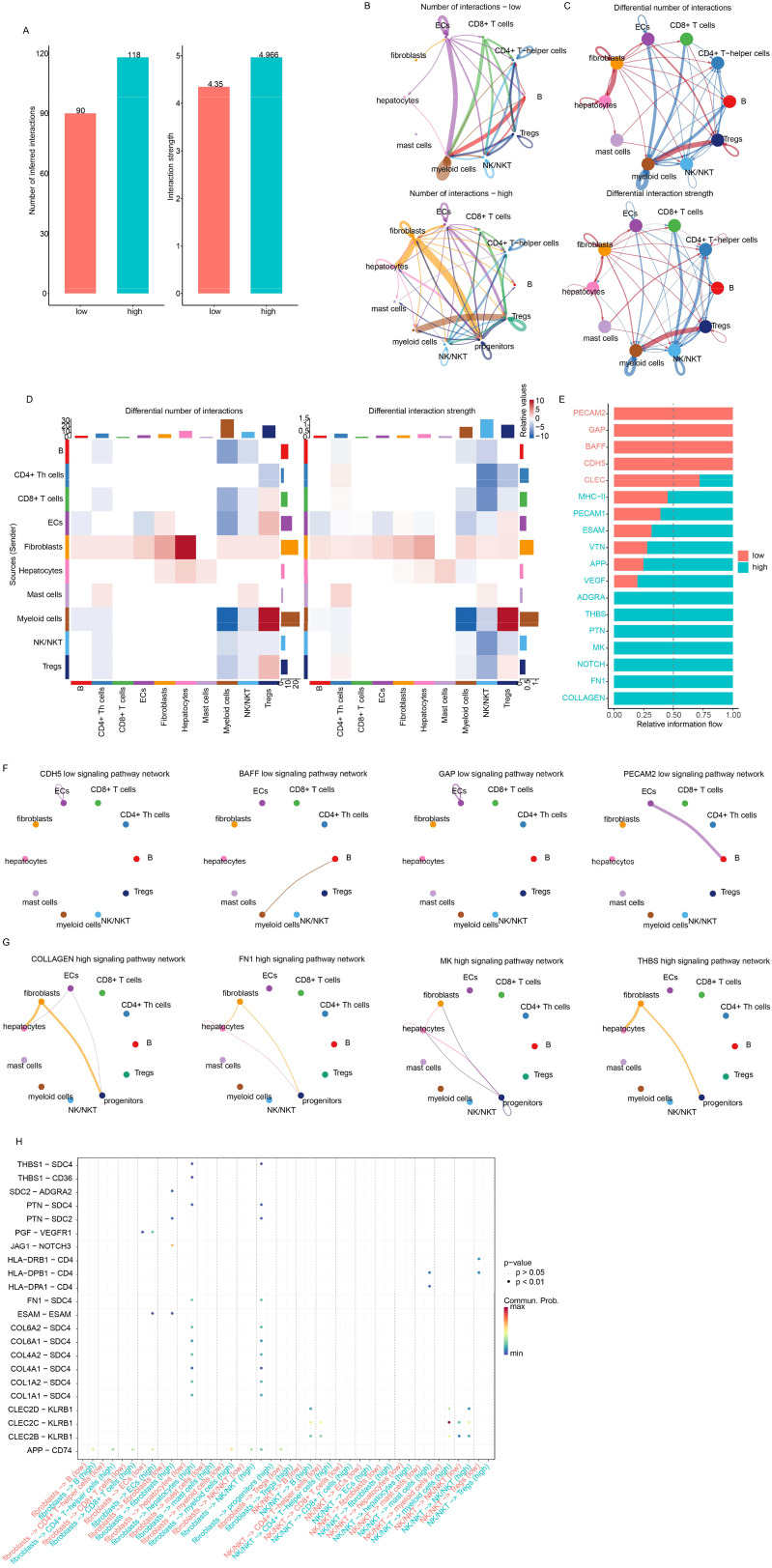
Drug sensitivity of low-/high-risk samples. **(A)** The overall interaction number and strength between high- and low-risk samples. **(B)** However, the detailed interactions of cell types differed. Differential interactions were visualized using circle plots **(C)** and heatmaps **(D)**. **(E)** Information flow analysis revealed differential pathways, primarily involving fibroblasts and NKT cells. Specific pathways in low- **(F)** and high-risk **(G)** samples were also visualized. Fibroblast/NK/NKT-related signaling with other cell types is also shown **(H)**.

Since the candidate genes were immune cell activation-related genes, the differences in cell–cell interactions reflected by the signature were investigated next. Overall, the interaction number and strength were similar between the high- and low-risk groups (**Figure 7A**), while the interactions between cell types were different (**Figure 7B**). The differential interactions among cell types were estimated and visualized in **Figures 7C, D**. The most altered interactions between groups were those involving fibroblasts, NKT cells, and myeloid cells. The differential pathways were also identified (**Figure 7E**). PECAM2, GAP, BAFF, and CDH5 were detected only in the low-risk samples, while COLLAGEN, FN1, NOTCH, THBS, ADGRA, VEGF, APP, and VTN signaling were detected specifically in the high-risk samples (**Figures 7F, G**). The differences in fibroblast interactions were mostly between mast cells and ECs, while the differences in NKT cell interactions were mostly between myeloid cells and mast cells (**Figure 7H**). Taken together, these results suggest that the signature reflects the status of immune cell–cell interactions, especially those involving fibroblasts, myeloid cells, and NK cells.

### Drug resistance and the signature

**Figure 8:**
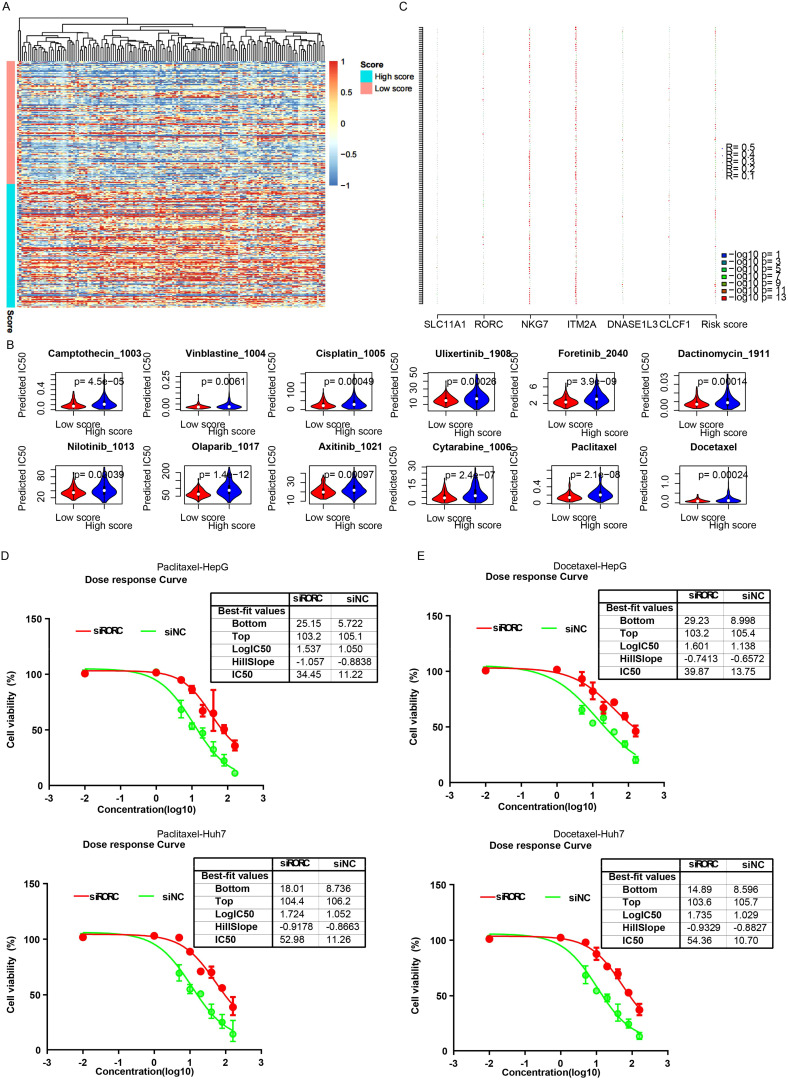
The signature and drug sensitivity. **(A)** Heatmap of differential drugs between low/high-risk samples. **(B)** Boxplots showing IC50 values for various drugs in high-risk and low-risk HCC groups, calculated using the “OncoPredict” algorithm. **(C)** Correlation analysis between candidate genes/signatures and drug IC50 values. Scatterplots reveal a significant positive correlation between RORC expression and the IC50 values of paclitaxel **(D)** and docetaxel **(E)**.

Since the signature reflected various HCC statuses and these statuses were significantly associated with drug response, the IC50 value of each drug for each cancer sample was evaluated using the “oncopredict” algorithm. Differences in IC50 values between the high-risk and low-risk groups for each drug were estimated. The low-risk samples were sensitive to most differential drugs (**Figures 8A, B**), while the high-risk samples were sensitive to only a few drugs. Correlations between candidate genes/the signature and drug IC50 values were evaluated, and NKG7 and ITM2A contributed the most to drug sensitivity (**Figure 8C**). Since it is difficult to alter the signature value, RORC was knocked down, and the drug sensitivity was quantified by estimating the IC50. As expected, the siRORC group was significantly more resistant to both paclitaxel and docetaxel in both the HepG2 and Huh7 cell lines (**Figures 8D, E**). Collectively, these results indicate that candidate gene RORC and the signature predict chemotherapy sensitivity, especially to paclitaxel and docetaxel.

### RORC inhibits the proliferation and migration of HCC cell lines

**Figure 9:**
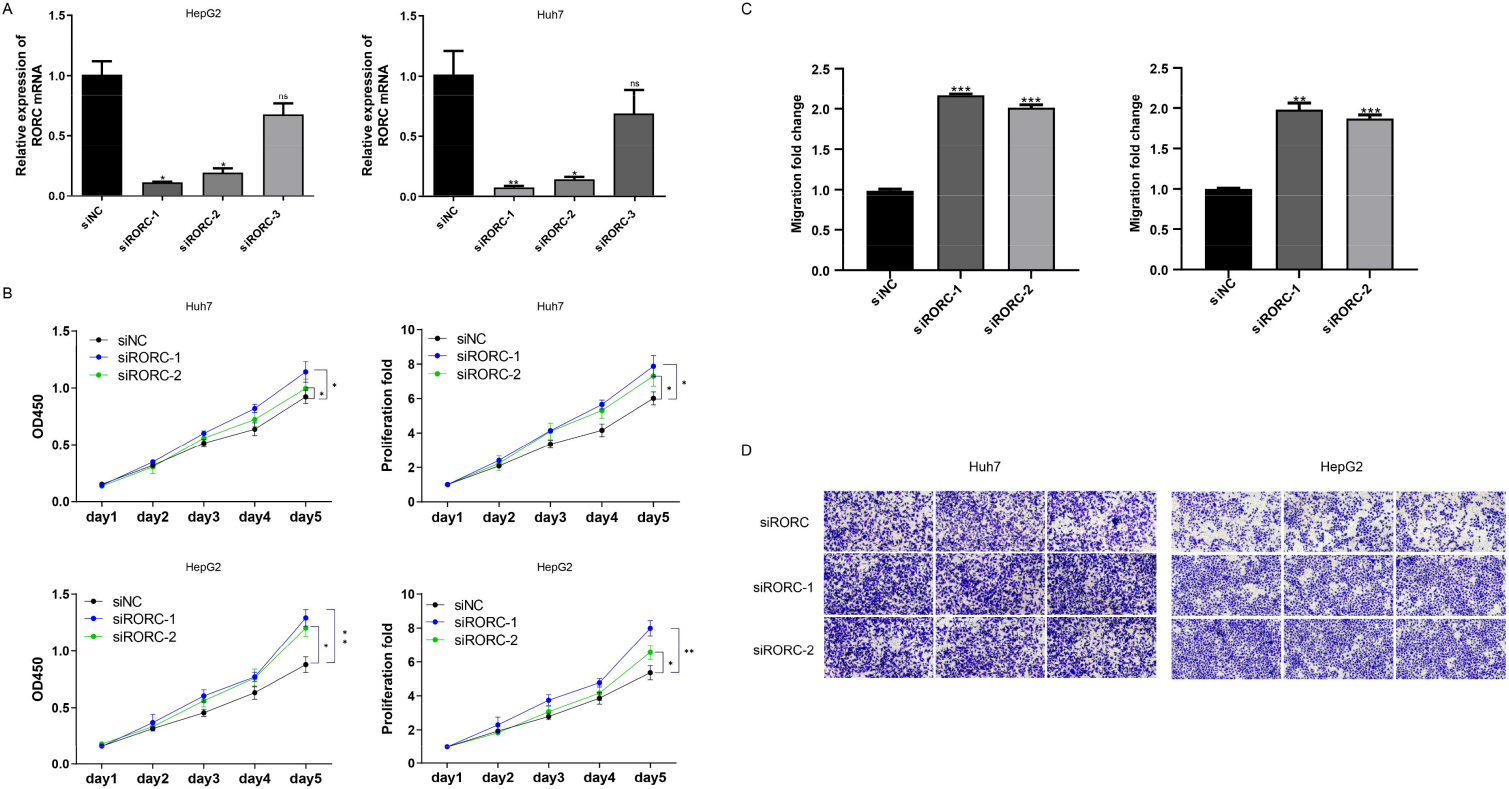
RORC and DNASE1L3 knockdown promote proliferation and migration. **(A)** Knockdown efficiency of RORC. **(B)** Knocking down RORC significantly enhanced the proliferation rate of the HCC cell lines HepG2 and Huh7. **(C, D)** Migration rate was also significantly upregulated after RORC was knocked down. HCC, hepatocellular carcinoma. *, p<0.05, **, p<0.01, ***, p<0.001. NS, Not Significant.

To validate the roles of the candidate genes, the growth of the HCC cell lines HepG2 and Huh7 was assessed following the knockdown of RORC (**Figure 9A**). The growth rate was measured using the CCK-8 assay. Significant changes in the proliferation of both cancer cell lines were observed (**Figure 9B**). Consistently, the downregulation of RORC also enhanced the migration ability of both cell lines (**Figures 9C, D**), indicating the role of RORC in inhibiting the proliferation and migration phenotypes of HCC cell lines.

### RORC suppresses CDC6 transcription to oppose proliferation of HCC cell lines

**Figure 10:**
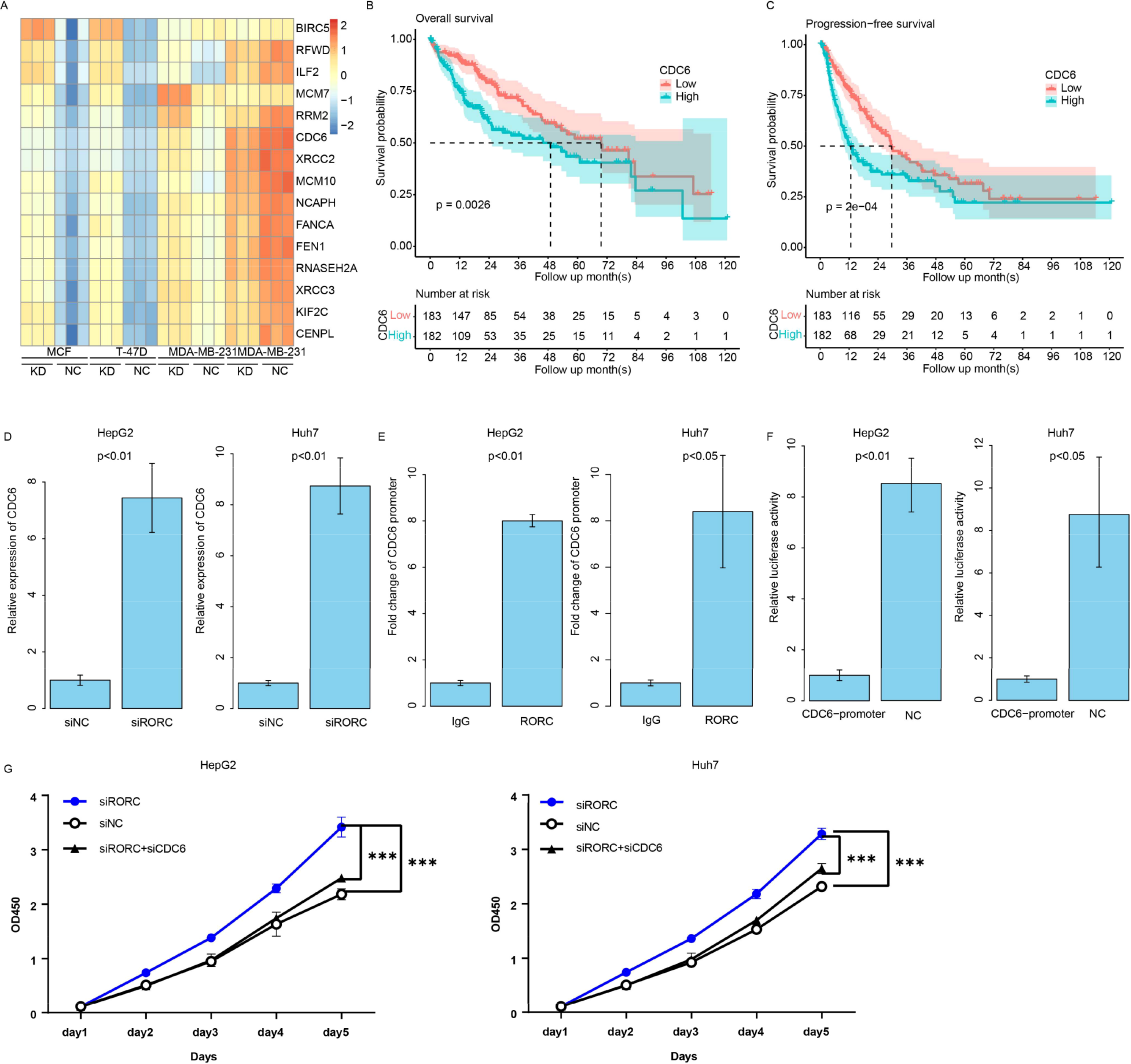
RORC suppresses proliferation in HCC by inhibiting CDC6. **(A)** Differentially expressed genes (DEGs) identified from RORC knockdown and overexpression analyses. Kaplan–Meier curves showing the association of CDC6 expression with overall survival in TCGA dataset using overall **(B)** and progression-free survival **(C)** in TCGA datasets. **(D)** Relative expression of CDC6 detected via qPCR upon RORC knockdown in both cell lines (p < 0.01). **(E)** ChIP-PCR showing RORC enrichment at the CDC6 promoter region. **(F)** Luciferase reporter assay using the CDC6 promoter. **(G)** CCK-8 proliferation assays in HepG2 and Huh7 cells after RORC and/or CDC6 knockdown (p < 0.001). HCC, hepatocellular carcinoma; ChIP, chromatin immunoprecipitation. '***', means p<0.001.

The RORC was reported as a transcription factor, while the detailed downstream genes are still not clear. To investigate the role of RORC in HCC, we analyzed several cell lines knocking down and/or overexpressing RORC, and we identified the differentially expressed genes in all these cell lines between control and overexpression groups (**Figure 10A**). Of these genes, CDC6 was the frequently reported prognostic gene in HCC. The expression of CDC6 was also significantly associated with survival in TCGA dataset (**Figures 10B, C**). To validate this finding, we knocked down RORC in the HepG2 and Huh7 cell lines and detected significantly increased CDC6 expression (**Figure 10D**). Additionally, ChIP-PCR revealed that RORC was enriched in the promoter region of CDC6 (**Figure 10E**), and the luciferase report indicated that the RORC promoter significantly decreased the luciferase signal intensity (**Figure 10F**). Since the role of CDC6 has been widely reported, we next investigated whether RORC inhibits proliferation via CDC6. After knocking down CDC6 in the siRORC group, the proliferation rate of the HepG2 and Huh7 cell lines was quantified using CCK-8 assay, as the previous result. CDC6 knockdown significantly restored the proliferation increase caused by RORC knockdown in both cell lines (**Figure 10F**). Taken together, these results indicate that RORC suppresses proliferation via CDC6 inhibition.

### RORC serves as a prognostic marker

**Figure 11:**
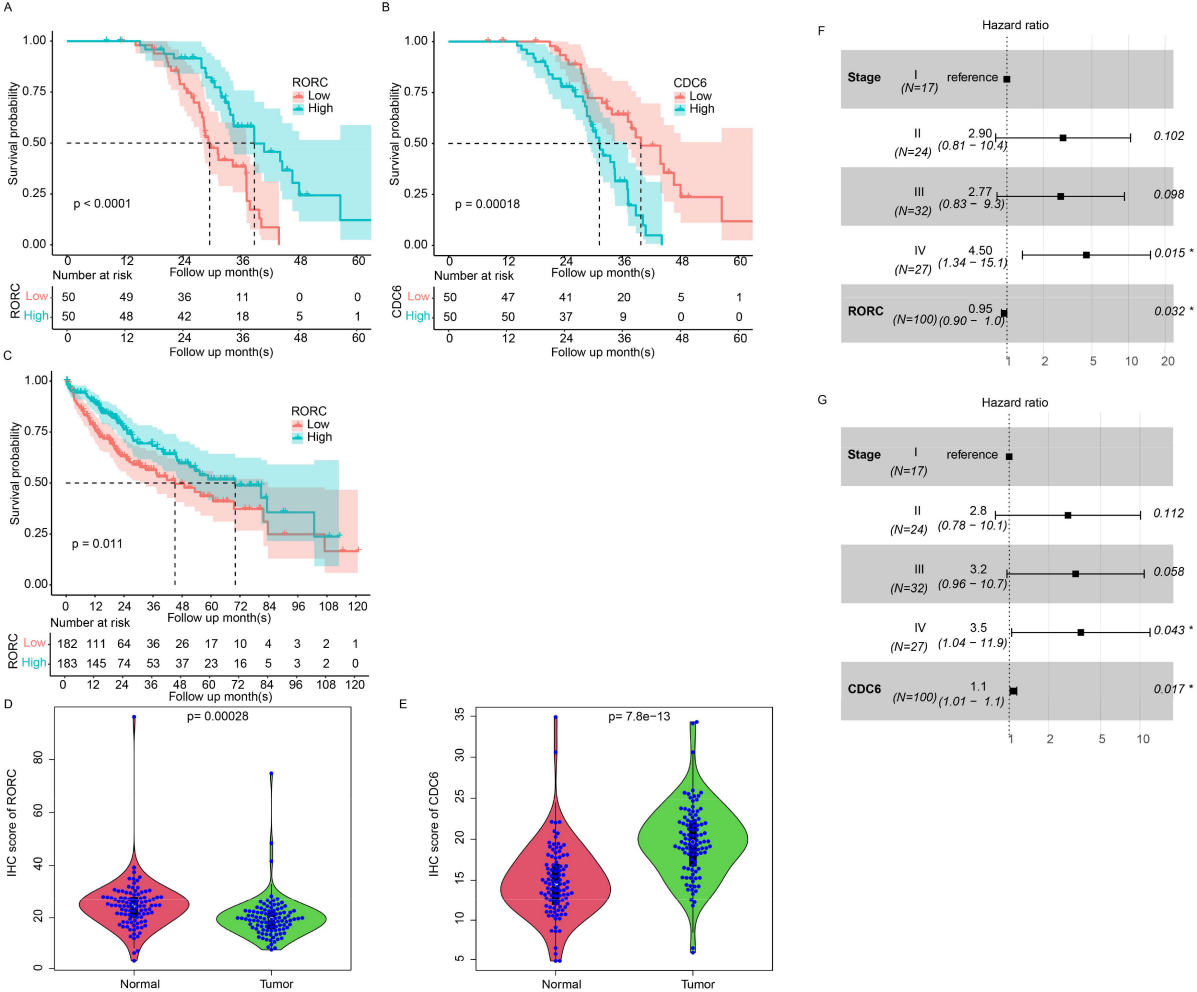
Prognostic value of RORC and CDC6 in HCC using IHC. Kaplan–Meier survival analyses of 100 primary HCC samples showing prolonged survival in patients with high RORC expression (**A**, p < 0.0001) and worse survival in patients with high CDC6 expression (**B**, p < 0.001). **(C)** TCGA dataset confirms that high RORC expression correlates with better overall survival. **(D, E)** Immunohistochemical staining microarray of 100 primary HCC samples reveals significantly lower RORC expression in tumor tissues compared to normal tissues (**D**, p < 0.001) and significantly higher CDC6 expression in tumor tissues (**E**, p < 7.8e−13). **(F, G)** Cox multivariate regression analysis identifies RORC **(G)** and CDC6 **(H)** as significant independent predictors of overall survival (p < 0.05) in IHC dataset. HCC, hepatocellular carcinoma; IHC, immunohistochemical.

To further address the prognostic role of RORC and CDC6, we collected 100 primary HCC samples from our affiliation and quantified the expression value with immune histochemical staining microarray. As a result, higher expression of RORC resulted in a prolonged survival period compared to the RORC-low group (**Figure 11A**), while elevated CDC6 was correlated with a worse survival rate (**Figure 11B**), which is consistent with TCGA dataset (**Figures 11C**, **10B, C**). It was noted the RORC was significantly downregulated in tumor tissues compared to the normal tissue (**Figure 11D**), while CDC6 was upregulated (**Figure 11E**). In addition, CDC6 and RORC were significantly associated with overall survival using Cox multivariate regression (**Figures 11F, G**, p < 0.05). Collectively, these results indicate that RORC and its downstream target CDC6 serve as prognostic markers for HCC.

## Discussion

As a chronic disease, HCC is characterized by long-term interactions between cancer cells and immune cells. During this process, the dynamic status of multiple cell types, including exhausted T cells (33), the complement system (34), and polarized macrophages (35), has been reported. Immune cell (especially T cell) activation is critical for cancer progression and treatment, especially for immunotherapy (36). However, activating the immune response in HCC involves multiple processes and involves various cell types. However, the detailed underlying mechanisms are still vague. To this end, we constructed a signature using immune response activation-related genes validated across datasets and investigated the heterogeneity reflected at multiple levels, including clinical, omics, drug response, and immune infiltration data. It is suspected that the heterogeneity of the model is a result of multiple gene-based signatures. For example, the candidate gene RORC was reported to regulate glycolysis and drug resistance in bladder cancer (37). A retrospective study revealed that SLC11A1 expression predicted the clinical outcome of glioma patients after immunotherapy (38). Another candidate gene, natural killer cell granule protein-7 (NKG7), is a well-known gene for activating the antitumor response of natural killer cells (39). NKG7 is also a therapeutic target for enhancing the efficacy of immunotherapy (40). Other candidate genes were also reported to be associated with drug response and immune infiltration (41, 42). As biomarkers, these single genes were not emphasized due to a lack of validation datasets, but their combination, the signature, was reproducible across cohorts. This indicates that the signature related to immune response activation-related genes is a valuable marker for HCC.

Among the immune cell types associated with the signature, we noticed that M0 macrophage was significantly enriched in the high-risk samples according to CIBERSORT. In consistent with estimated depleted CD8+ T cells in the high-risk samples, M0 macrophage enriched usually indicates an immune suppressive microenvironment, accompanied by worse survival (43, 44). Previous studies also developed HCC signatures using M0 macrophage-related genes and achieved good performance (45, 46). In addition, a higher macrophage-to-lymphocyte ratio also may influence the immunotherapy of melanoma (47). However, it is still not clear how the accumulation of M0 in tumor tissue affects the prognosis and treatment outcome.

In addition, during the cell–cell interaction analyses, we revealed that both high and low risk possess their specific signaling pathways. COLLAGEN, FN1, NOTCH, THBS, ADGRA, VEGF, APP, and VTN pathways were specific in the high-risk samples, while PECAM2, GAP, BAFF, and CDH5 were detected in only the low-risk samples. It is suspected that drugs targeting different pathways may benefit different patients. For example, drugs targeting VEGF including bevacizumab and regorafenib may benefit the high-risk samples, as well as NOTCH inhibitors. Drugs or monoclonal antibodies targeting PECAM2 GAP, BAFF, or CDH5 may benefit low-risk patients. Although the impact of some drugs in treating HCC is still not clear, the study may provide insight into personalized medicine in future drug development and HCC population segregation.

In this work, our results indicated that the candidate gene RORC in the signature regulates CDC6 by binding to the promoter region of CDC6 using ChIP-seq, siRNA, and luciferase assay. Instead of functioning as a transcription factor, it suppresses the expression of CDC6. Although studies regarding this gene have been reported, including cancer therapy (48), the detailed mechanism is still not clear. We revealed that it functions as a transcription factor to repress the expression of CDC6, which may provide new insight into investigating the role of RORC.

The preliminary limitation of this study is that it is a retrospective study, and the detailed sample information and treatment regimen are unknown, which makes it difficult to evaluate the bias of the signature among subgroups and the clinical treatment response. Although the IC50 was estimated using a prediction algorithm, the results are still less convincing and lack real-world results. Another limitation is that the validation dataset was generated from different platforms. Although the robustness of these methods has been verified, it is difficult to optimize the cutoff values for clinical use. Finally, the detailed functions of candidate genes and their role in immune cell activation are not clear, which makes it difficult to use candidate genes as potential therapeutic targets.

## Data Availability

The datasets presented in this study can be found in online repositories. The names of the repository/repositories and accession number(s) can be found in the article/supplementary material.

## References

[1] Foerster F Gairing SJ Ilyas SI Galle PR. Emerging immunotherapy for HCC: A guide for hepatologists. Hepatology. (2022) 75:1604–26. doi: 10.1002/hep.32447 PMC9117522 35253934

[2] Llovet JM Kelley RK Villanueva A Singal AG Pikarsky E Roayaie S. Hepatocellular carcinoma. Nat Rev Dis Primers. (2021) 7:6. doi: 10.1038/s41572-020-00240-3 33479224 PMC13537433

[3] Tang W Chen Z Zhang W Cheng Y Zhang B Wu F. The mechanisms of sorafenib resistance in hepatocellular carcinoma: theoretical basis and therapeutic aspects. Signal Transduct Target Ther. (2020) 5:87. doi: 10.1038/s41392-020-0187-x 32532960 PMC7292831

[4] Sperandio RC Pestana RC Miyamura BV Kaseb AO. Hepatocellular carcinoma immunotherapy. Annu Rev Med. (2022) 73:267–78. doi: 10.1146/annurev-med-042220-021121 34606324

[5] Dhanasekaran R. Deciphering tumor heterogeneity in hepatocellular carcinoma (HCC)-multi-omic and singulomic approaches. Semin Liver Dis. (2021) 41:9–18. doi: 10.1055/s-0040-1722261 33764481 PMC8136683

[6] Behrens S Wang XW. Dissecting intratumor heterogeneity in HCC: new research strategies and clinical implications. Carcinogenesis. (2022) 43:1103–9. doi: 10.1093/carcin/bgac099 PMC10122425 36512331

[7] Zhou Q Li L Sha F Lei Y Tian X Chen L. PTTG1 reprograms asparagine metabolism to promote hepatocellular carcinoma progression. Cancer Res. (2023) 83:2372–86. doi: 10.1158/0008-5472.CAN-22-3561 37159932

[8] Gu Z Wang L Dong Q Xu K Ye J Shao X. Aberrant LYZ expression in tumor cells serves as the potential biomarker and target for HCC and promotes tumor progression via csGRP78. Proc Natl Acad Sci U S A. (2023) 120:e2215744120. doi: 10.1073/pnas.2215744120 37428911 PMC10629575

[9] Yang YC Chien MH Lai TC Tung MC Jan YH Chang WM. Proteomics-based identification of TMED9 is linked to vascular invasion and poor prognoses in patients with hepatocellular carcinoma. J BioMed Sci. (2021) 28:29. doi: 10.1186/s12929-021-00727-5 33888099 PMC8063382

[10] Ma Q Jiang H Ma L Zhao G Xu Q Guo D. The moonlighting function of glycolytic enzyme enolase-1 promotes choline phospholipid metabolism and tumor cell proliferation. Proc Natl Acad Sci U S A. (2023) 120:e2209435120. doi: 10.1073/pnas.2209435120 37011206 PMC10104498

[11] Liu X Song Y Cheng P Liang B Xing D. Targeting HER2 in solid tumors: Unveiling the structure and novel epitopes. Cancer Treat Rev. (2024) 130:102826. doi: 10.1016/j.ctrv.2024.102826 39270365

[12] van ‘t Veer LJ Dai H van de Vijver MJ He YD Hart AA Mao M. Gene expression profiling predicts clinical outcome of breast cancer. Nature. (2002) 415:530–6. doi: 10.1038/415530a 11823860

[13] Cardoso F van’t Veer LJ Bogaerts J Slaets L Viale G Delaloge S. 70-gene signature as an aid to treatment decisions in early-stage breast cancer. N Engl J Med. (2016) 375:717–29. doi: 10.1056/NEJMoa1602253 27557300

[14] Davey MG Richard V Lowery AJ Kerin MJ. OncotypeDX^©^ Recurrence Score in BRCA mutation carriers: a systematic review and meta-analysis. Eur J Cancer. (2021) 154:209–16. doi: 10.1016/j.ejca.2021.06.032 34284256

[15] Chi H Zhao S Yang J Gao X Peng G Zhang J. T-cell exhaustion signatures characterize the immune landscape and predict HCC prognosis via integrating single-cell RNA-seq and bulk RNA-sequencing. Front Immunol. (2023) 14:1137025. doi: 10.3389/fimmu.2023.1137025 37006257 PMC10050519

[16] Deng M Sun S Zhao R Guan R Zhang Z Li S. The pyroptosis-related gene signature predicts prognosis and indicates immune activity in hepatocellular carcinoma. Mol Med. (2022) 28:16. doi: 10.1186/s10020-022-00445-0 35123387 PMC8818170

[17] Hanahan D Weinberg RA. Hallmarks of cancer: the next generation. Cell. (2011) 144:646–74. doi: 10.1016/j.cell.2011.02.013 21376230

[18] Ruiz de Galarreta M Bresnahan E Molina-Sánchez P Lindblad KE Maier B Sia D. β-catenin activation promotes immune escape and resistance to anti-PD-1 therapy in hepatocellular carcinoma. Cancer Discov. (2019) 9:1124–41. doi: 10.1158/2159-8290.CD-19-0074 PMC6677618 31186238

[19] Goldman MJ Craft B Hastie M Repečka K McDade F Kamath A. Visualizing and interpreting cancer genomics data via the Xena platform. Nat Biotechnol. (2020) 38:675–8. doi: 10.1038/s41587-020-0546-8 PMC7386072 32444850

[20] Cerami E Gao J Dogrusoz U Gross BE Sumer SO Aksoy BA. The cBio cancer genomics portal: an open platform for exploring multidimensional cancer genomics data. Cancer Discov. (2012) 2:401–4. doi: 10.1158/2159-8290.CD-12-0095 PMC3956037 22588877

[21] Liberzon A Birger C Thorvaldsdóttir H Ghandi M Mesirov JP Tamayo P. The Molecular Signatures Database (MSigDB) hallmark gene set collection. Cell Syst. (2015) 1:417–25. doi: 10.1016/j.cels.2015.12.004 PMC4707969 26771021

[22] Skidmore ZL Wagner AH Lesurf R Campbell KM Kunisaki J Griffith OL. GenVisR: genomic visualizations in R. Bioinformatics. (2016) 32:3012–4. doi: 10.1093/bioinformatics/btw325 PMC5039916 27288499

[23] Yu G Wang LG Han Y He QY. clusterProfiler: an R package for comparing biological themes among gene clusters. Omics. (2012) 16:284–7. doi: 10.1089/omi.2011.0118 PMC3339379 22455463

[24] Aran D Hu Z Butte AJ. xCell: digitally portraying the tissue cellular heterogeneity landscape. Genome Biol. (2017) 18:220. doi: 10.1186/s13059-017-1349-1 29141660 PMC5688663

[25] Newman AM Liu CL Green MR Gentles AJ Feng W Xu Y. Robust enumeration of cell subsets from tissue expression profiles. Nat Methods. (2015) 12:453–7. doi: 10.1038/nmeth.3337 PMC4739640 25822800

[26] Li T Fu J Zeng Z Cohen D Li J Chen Q. TIMER2.0 for analysis of tumor-infiltrating immune cells. Nucleic Acids Res. (2020) 48:W509–w514. doi: 10.1093/nar/gkaa407 32442275 PMC7319575

[27] Finotello F Mayer C Plattner C Laschober G Rieder D Hackl H. Molecular and pharmacological modulators of the tumor immune contexture revealed by deconvolution of RNA-seq data. Genome Med. (2019) 11:34. doi: 10.1186/s13073-019-0638-6 31126321 PMC6534875

[28] Racle J de Jonge K Baumgaertner P Speiser DE Gfeller D. Simultaneous enumeration of cancer and immune cell types from bulk tumor gene expression data. Elife. (2017) 6. doi: 10.7554/eLife.26476 PMC5718706 29130882

[29] Maeser D Gruener RF Huang RS. oncoPredict: an R package for predicting *in vivo* or cancer patient drug response and biomarkers from cell line screening data. Brief Bioinform. (2021) 22. doi: 10.1093/bib/bbab260 PMC8574972 34260682

[30] Sharma A Seow JJW Dutertre CA Pai R Blériot C Mishra A. Onco-fetal reprogramming of endothelial cells drives immunosuppressive macrophages in hepatocellular carcinoma. Cell. (2020) 183:377–394.e21. doi: 10.1016/j.cell.2020.08.040 32976798

[31] Gribov A Sill M Lück S Rücker F Döhner K Bullinger L. SEURAT: visual analytics for the integrated analysis of microarray data. BMC Med Genomics. (2010) 3:21. doi: 10.1186/1755-8794-3-21 20525257 PMC2893446

[32] Jin S Guerrero-Juarez CF Zhang L Chang I Ramos R Kuan CH. Inference and analysis of cell-cell communication using CellChat. Nat Commun. (2021) 12:1088. doi: 10.1038/s41467-021-21246-9 33597522 PMC7889871

[33] Barsch M Salié H Schlaak AE Zhang Z Hess M Mayer LS. T-cell exhaustion and residency dynamics inform clinical outcomes in hepatocellular carcinoma. J Hepatol. (2022) 77:397–409. doi: 10.1016/j.jhep.2022.02.032 35367533

[34] Qian X Yang Z Gao L Liu Y Yan J. The role of complement in the clinical course of hepatocellular carcinoma. Immun Inflammation Dis. (2022) 10:e569. doi: 10.1002/iid3.v10.3 PMC8926509 34813686

[35] Tang B Zhu J Wang Y Chen W Fang S Mao W. Targeted xCT-mediated Ferroptosis and Protumoral Polarization of Macrophages Is Effective against HCC and Enhances the Efficacy of the Anti-PD-1/L1 Response. Adv Sci (Weinh). (2023) 10:e2203973. doi: 10.1002/advs.202203973 36442849 PMC9839855

[36] Sangro B Sarobe P Hervás-Stubbs S Melero I. Advances in immunotherapy for hepatocellular carcinoma. Nat Rev Gastroenterol Hepatol. (2021) 18:525–43. doi: 10.1038/s41575-021-00438-0 PMC8042636 33850328

[37] Cao D Qi Z Pang Y Li H Xie H Wu J. Retinoic acid-related orphan receptor C regulates proliferation, glycolysis, and chemoresistance via the PD-L1/ITGB6/STAT3 signaling axis in bladder cancer. Cancer Res. (2019) 79:2604–18. doi: 10.1158/0008-5472.CAN-18-3842 30808674

[38] Xu H Zhang A Fang C Zhu Q Wang W Liu Y. SLC11A1 as a stratification indicator for immunotherapy or chemotherapy in patients with glioma. Front Immunol. (2022) 13:980378. doi: 10.3389/fimmu.2022.980378 36531992 PMC9748290

[39] Li XY Corvino D Nowlan B Aguilera AR Ng SS Braun M. NKG7 is required for optimal antitumor T-cell immunity. Cancer Immunol Res. (2022) 10:154–61. doi: 10.1158/2326-6066.CIR-20-0649 35013002

[40] Wen T Barham W Li Y Zhang H Gicobi JK Hirdler JB. NKG7 is a T-cell-intrinsic therapeutic target for improving antitumor cytotoxicity and cancer immunotherapy. Cancer Immunol Res. (2022) 10:162–81. doi: 10.1158/2326-6066.CIR-21-0539 PMC8816890 34911739

[41] Deng Z Xiao M Du D Luo N Liu D Liu T. DNASE1L3 as a prognostic biomarker associated with immune cell infiltration in cancer. Onco Targets Ther. (2021) 14:2003–17. doi: 10.2147/OTT.S294332 PMC7987320 33776450

[42] Zhang Z Tan X Luo J Yao H Si Z Tong JS. The miR-30a-5p/CLCF1 axis regulates sorafenib resistance and aerobic glycolysis in hepatocellular carcinoma. Cell Death Dis. (2020) 11:902. doi: 10.1038/s41419-020-03123-3 33097691 PMC7584607

[43] Farha M Jairath NK Lawrence TS El Naqa I. Characterization of the tumor immune microenvironment identifies M0 macrophage-enriched cluster as a poor prognostic factor in hepatocellular carcinoma. JCO Clin Cancer Inform. (2020) 4:1002–13. doi: 10.1200/CCI.20.00077 PMC7713549 33136432

[44] Zhang Z Wang Z Huang Y. Comprehensive analyses of the infiltrating immune cell landscape and its clinical significance in hepatocellular carcinoma. Int J Gen Med. (2021) 14:4695–704. doi: 10.2147/IJGM.S326535 PMC8384430 34447264

[45] Zhang Y Zou J Chen R. An M0 macrophage-related prognostic model for hepatocellular carcinoma. BMC Cancer. (2022) 22:791. doi: 10.1186/s12885-022-09872-y 35854246 PMC9294844

[46] You JA Gong Y Wu Y Jin L Chi Q Sun D. WGCNA, LASSO and SVM algorithm revealed RAC1 correlated M0 macrophage and the risk score to predict the survival of hepatocellular carcinoma patients. Front Genet. (2021) 12:730920. doi: 10.3389/fgene.2021.730920 35493265 PMC9044718

[47] Jairath NK Farha MW Jairath R Harms PW Tsoi LC Tejasvi T. Prognostic value of intratumoral lymphocyte-to-monocyte ratio and M0 macrophage enrichment in tumor immune microenvironment of melanoma. Melanoma Manag. (2020) 7:Mmt51. doi: 10.2217/mmt-2020-0019 PMC7727784 33318782

[48] He S Yu J Sun W Sun Y Tang M Meng B. A comprehensive pancancer analysis reveals the potential value of RAR-related orphan receptor C (RORC) for cancer immunotherapy. Front Genet. (2022) 13:969476. doi: 10.3389/fgene.2022.969476 36186454 PMC9520743

